# Fourier Transform Mass Spectrometry: The Transformation of Modern Environmental Analyses

**DOI:** 10.3390/ijms17010104

**Published:** 2016-01-14

**Authors:** Lucy Lim, Fangzhi Yan, Stephen Bach, Katianna Pihakari, David Klein

**Affiliations:** 1 Department of Environmental Toxicology, Texas Tech University, Lubbock, TX 79416, USA; lucy.lim@ttu.edu; 2 Department of Chemistry, University of Texas at San Antonio, San Antonio, TX 78249, USA; fangzhiy@gmail.com (F.Y.); stephen.bach@utsa.edu (S.B.); 3 Thermo Fisher Scientific, Waltham, MA 02451, USA; katianna.pihakari@thermofisher.com

**Keywords:** mass spectrometry, environmental, high resolution, Orbitrap, FTMS

## Abstract

Unknown compounds in environmental samples are difficult to identify using standard mass spectrometric methods. Fourier transform mass spectrometry (FTMS) has revolutionized how environmental analyses are performed. With its unsurpassed mass accuracy, high resolution and sensitivity, researchers now have a tool for difficult and complex environmental analyses. Two features of FTMS are responsible for changing the face of how complex analyses are accomplished. First is the ability to quickly and with high mass accuracy determine the presence of unknown chemical residues in samples. For years, the field has been limited by mass spectrometric methods that were based on knowing what compounds of interest were. Secondly, by utilizing the high resolution capabilities coupled with the low detection limits of FTMS, analysts also could dilute the sample sufficiently to minimize the ionization changes from varied matrices.

## 1. Introduction

Toxic environmental chemical sources continue to be a problematic global health concern. There are multiple sources of pollutants which include agricultural, industrial, as well as other point sources such as mining, foundries and smelters, along with other metal-based industrial operations [1]. Excessive levels of pesticides in agricultural products and in herbal medicines are also a concern with the public involved in healthcare [2]. Wastewater sludge that contains acidic contaminants is a growing area of concern as well [3]. These complex solid-liquid phase matrices require advanced high performance methods to monitor ultra-trace (part per trillion) levels of target compounds. Some of these have been detected even after the final purification stage in drinking water [4]. The presence of un-metabolized pharmaceuticals discharged into wastewater also is unavoidable with the current treatment processes in place [5]. Metal-based nanoparticles, which are an emerging pollutant stream, have been determined in the environment by inductively coupled plasma-mass spectrometry, for example [6]. The threat posed by nanoparticle pollution has become a difficult 21st century analytical problem. Diesel-burning engines are known to emit nanoparticles and these have been analyzed by thermal desorption particle beam mass spectrometry [7].

Fundamentally, environmental analyses consist of obtaining a representative sample, extracting the compound(s) of interest, performing appropriate sample clean-up, and deriving the concentration of the extract and determination of the compound identity and/or quantity. The United States Environmental Protection Agency (EPA) has collected resources including many environmental chemistry methods (ECMs) [8]. Environmental sample analyses typically have interference effects that result in poor quantitative data. Mass spectrometry requires that ions be produced in the gas phase. This may lead to sample preparation requiring extraction of the analyte from the matrix material or using matrix-matched calibration to compensate for matrix interferents. The preparation step results in diluting the analyte and thereby reducing the chances of recovering all trace chemical compound(s) of interest [9,10]. Another issue with sample preparation is that matrix extraction may change the analyte’s ionization efficiency, directly impacting detection limits [11]. The change may result in signal suppression or enhancement. Differences in ionization may also occur due to the presence of solvent or matrix interference [9,12]. Cost-effectiveness of sample analysis is a significant issue for environmental applications of mass spectrometry. Due to the complexity of many environmental samples, time-consuming and labor-intensive preparation steps are required to extract the compound of primary interest. This limited early investigations to targeted inquiries of study material [13].

Analytical ECMs have followed the improvements in mass spectrometers over the last decades. Initially, determinations were made with gas chromatography (GC) or liquid chromatography (LC) with specific detectors. These diverse detectors were rapidly replaced when mass spectrometry (MS) was mated to chromatographic separations in the hyphenated techniques of gas chromatography-mass spectrometry (GC-MS) and liquid chromatography-mass spectrometry (LC-MS). While GC-MS is still the workhorse for many environmental analyses, it is limited to compounds that can be volatilized without decomposition and are below 500 Da. It was recently noted that only organochlorine pesticides had better performance on GC-MS with all other classes of pesticides having wider scope and better sensitivity with LC-MS [14]. LC-MS has become the method of choice for many environmental applications.

Even though LC-MS has become a standard technique in many bio-related applications, it is recognized that a GC with 30 m of fused silica column provides much higher chromatographic resolution for small molecules. Various MS methods were employed in an attempt to make up for the lack of separating power of the shorter liquid chromatography columns. Chief among these methods was the use of tandem mass spectrometry (MS/MS or MS^2^). Early ion traps were quickly adapted to perform MS/MS [15]. These MS/MS methods could now be used to determine a compound in a complex matrix by isolating the target ion of a compound followed by MS/MS to generate product ions confirming the identity of the target analyte. By interfacing chromatography with MS/MS, specificities and sensitivities achieved were equivalent to radioimmunoassay and GC-MS [16]. This initiated a shift in research studies from target-oriented analyses to full scan mass spectrometry that has the capability to produce a complete mass spectrum of target molecules as well as determine molecules in unknown mixtures [17,18]. This was initially seen as the answer to analyzing complex environmental samples. However, it has been reported that these low resolution mass spectrometers were challenged when analyzing complex samples because of their low mass accuracy. For instance, in the determination of perfluorooctane sulfonate, an endogenous compound (cholate) has the same nominal mass for the precursor ion and product ions [19]. In these cases, an investigator can either perform better chromatography to separate the compound of interest from interferents or employ a mass spectrometer with a mass accuracy in the low ppm range. This MS approach is not always the answer, but has been investigated [20].

A MS review whose focus is on environmental applications is done biennially in Analytical Chemistry [21]. The current perspective is focused on high resolution Fourier transform mass spectrometry (FTMS) instrumentation that has accurate mass capabilities below 10 ppm. The high cost limits the availability of the Fourier transform ion cyclotron resonance (FT-ICR) MS instrumentation to many applications. The Orbitrap technology, on the other hand, has been increasing in popularity because of its lower cost and lack of cryogens, and the vast majority of current work has been performed using the Orbitrap platform for the analysis of demanding environmental applications. Therefore, this perspective will mainly focus on the Orbitrap.

## 2. Fourier Transform Ion Cyclotron Resonance (FT-ICR)

**Figure 1:**
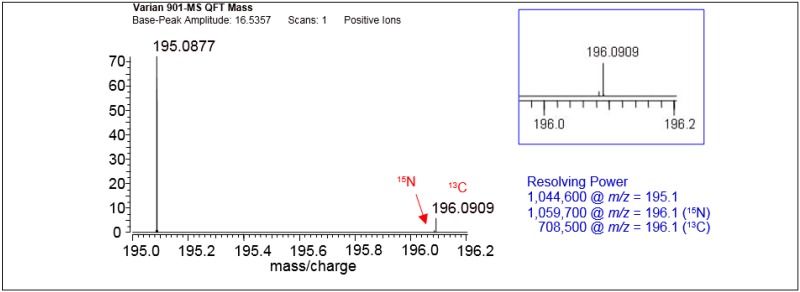
A high resolution spectrum of caffeine analyzed with a Varian 901-MS-QFT (Mass spectrometry-Quadrupole-Fourier Transform) mass spectrometer. The instrument was equipped with an electrospray ionization source (ZSpray) and a 9.4 T superconducting magnet.

Fourier transform ion cyclotron resonance (FT-ICR) mass spectrometers are the gold standard because of their high resolution and their high accurate mass capability [22]. These ultra-high resolution instruments enable the separation of isobaric species using their high accurate mass capabilities. This can be seen in Figure 1 which illustrates the low abundance ^13^C (^13^CC_7_H_10_N_4_O_2_) and ^15^N (C_8_H_10_N_3_^15^NO_2_) isotopes resolved for caffeine (C_8_H_10_N_4_O_2_).

FT-ICRs are very expensive instruments that require liquid helium-cooled superconducting magnets in order to operate. The high cost has kept these instruments only in advanced research laboratories and some academic settings. While it is clear that the mass resolution and the mass accuracy of these instruments is unsurpassed, the Orbitrap provides a more accessible and affordable avenue to high resolution and high mass accuracy. The usefulness of an Orbitrap has been clearly stated by Makarov *et al.* [23]: “These levels of resolving power [of the Orbitrap] are still far below, and will remain below record values obtained in FT-ICR, but it is more than adequate even for most demanding complex mixtures such as petroleum or humic acids”.

## 3. Orbitrap

**Figure 2:**
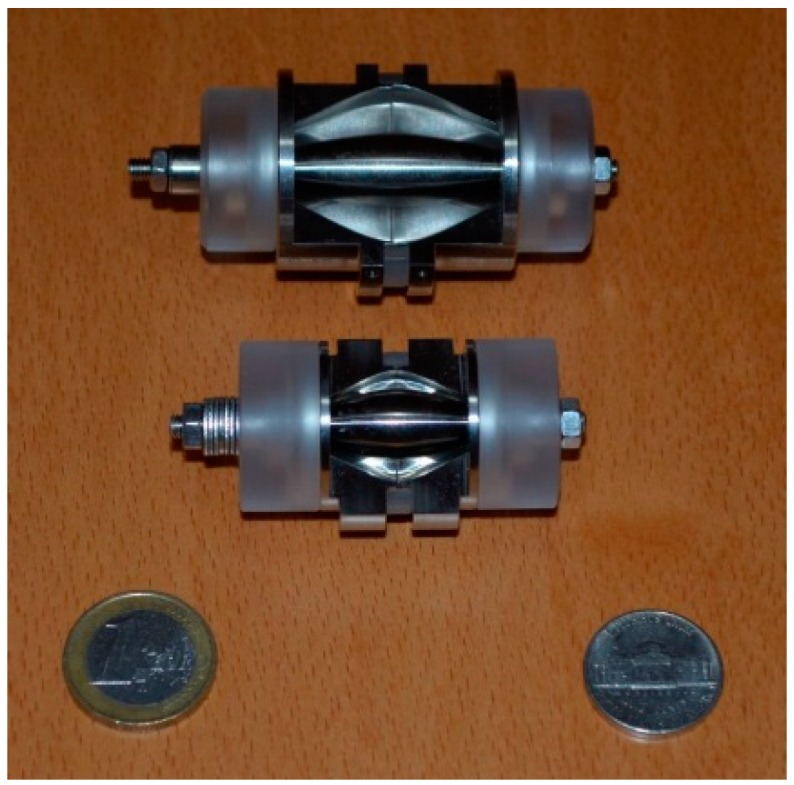
Two Orbitrap analyzers with both a 1-Euro coin and an American nickel as a reference for size [24]. The smaller device is the newer ultra-high resolution Orbitrap analyzer.

The Orbitrap technology is a more widely available technology for high resolution MS (HRMS) with a high accuracy mass. Figure 2 shows two Orbitrap analyzers with both European and American coins as a reference for size. The smaller device is the newer ultra-high resolution Orbitrap analyzer.

**Figure 3:**
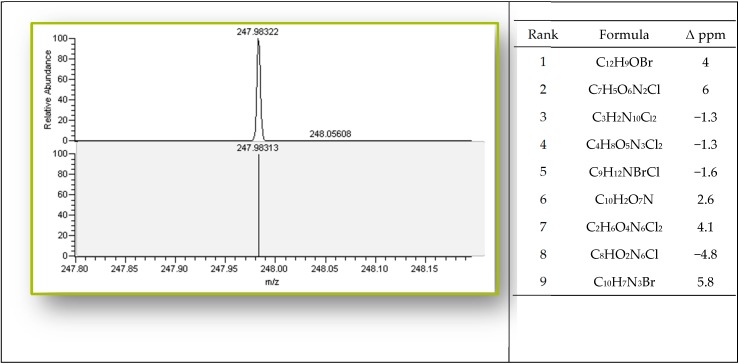
The importance of high mass accuracy: The high mass accuracy allows researchers to limit the number of possible molecular formulae significantly based on the accurate mass measurement. Here the correct formula, C_12_H_9_OBr (1-bromo-4-phenoxy-benzene), is the highest ranked formula based on lowest mass error. While other molecular formulas are possible they can be excluded when combined with knowledge of the target ion. Used with permission from Thermo Scientific; Waltham, MA, USA.

The challenge in MS performance is measuring the *m*/*z* of a composition of ions across a wide molecular range. One option is to perform targeted analysis of a specific chemical, which is commonly done by using LC-MS/MS on triple-quadrupole mass spectrometers [25] Another approach is to implement untargeted analyses [26]. This “shotgun” method is an advanced development in MS to identify and quantify all the compounds in a sample simultaneously. Orbitrap-based instruments with their higher sensitivity than more common quadrupole instruments can perform analysis on samples at greater dilutions to minimize the background interference. This mass spectrometer can be utilized for complex endogenous, exogenous or xenometabolite matrix samples [27,28,29,30]. For compounds mimicking environmentally relevant analytes, Figure 3 demonstrates the mass measurement accuracy and high resolutions attainable by these analyzers.

The Orbitrap is considered by many users as a robust analyzer that provides quantitative data on both clinical and environmental molecular biomarkers [31]. The Orbitrap has been typically mated to LC [32], and coupling with GC was introduced recently [33]. The ionization options for LC-MS include electrospray ionization (ESI), nanospray (nESI), atmospheric pressure chemical ionization (APCI) and atmospheric pressure photoionization (APPI), while for the GC-MS instrument the available ionization is either electron impact (EI) or chemical ionization (CI).

Descriptions of the Orbitrap and its operational theory are fully covered in prior work [34]. Once the ions are transferred into the Orbitrap analyzer, the ions are trapped in an electrostatic field created by both the spindle and outer electrodes [35,36]. The trapped ions move radially and oscillate along a central spindle (Orbitrap) electrode. The mass accuracy of the Orbitrap instrument is 2–5 ppm [37]. In the current models, <1 ppm mass accuracy is obtained with internal calibration while for external calibration the mass accuracy is specified as <3 ppm. The mass resolution of the classic Orbitrap analyzer can be up to 150,000 while the new ultra-high field Orbitrap analyzers have resolving power of more than 600,000 [23].

The ion traps of hybrid instruments are also capable of performing multiple MS steps, which aids in elucidating important structural information [38]. A search of case studies utilizing this technology reveals that there are applications for emerging environmental contaminants [39].

### Selected Environmental Applications of the Orbitrap

A single-stage Orbitrap-MS was used to identify and quantify non-targeted analytes in a U.S. Food and Drug Administration (FDA) study testing dog food. These results were compared to a targeted method based on a triple-quadrupole LC-MS/MS [40]. The biomonitoring of pesticides in urine [41] and the confirmatory analysis for growth-promoting agents in meat production [42] has accurately been determined using Orbitrap high resolution MS analyzers.

The identification of fullerenes in wastewater matrices was determined and quantified using an Orbitrap [43,44]. This technology enabled fewer sampling repetitions which allowed for an increased sample throughput and less wasted sample [42]. Optimization of matrix effects from sewage water (influent and effluent) improved the detection of metabolites and showed satisfactory recovery and precision for most known compounds [28,45,46]. Additional examples of successful screening using an Orbitrap include the determination of pesticide levels in soils and food [47] and various plant and fungal metabolites discovered in animal feed [48].

The elucidation of structural fragments without any pre-treatment was reported for metabolomic analysis of green and black tea extracts using an LTQ Orbitrap XL (a hybrid linear ion trap Orbitrap mass spectrometer) [49], and for direct analysis of red wine using ultra-fast chromatography and high resolution MS [50]. Rapid screening of textile samples for 19 human health and environmental toxins using a LTQ Orbitrap achieved a limit of quantitation (LOQ) lower than the adopted European Union regulations [51].

The level of pharmaceutical metabolites and parent compounds in surface waters has increased in both treated waste water and drinking water. This problem was addressed when a LTQ Orbitrap was used to provide better data for these complex matrices [52]. Mycotoxins and pesticide residues in agricultural products have become a serious human health concern. A high-throughput method was developed using an Orbitrap for high resolution MS after a single-stage extraction in spice analysis [53].

The emerging use of nanotechnology to enhance the properties of a variety of products has potential negative impacts to health and safety. In order to adequately evaluate the environmental risks requires further study of these nanomaterials using advanced mass spectrometry–based analytical techniques [54]. Screening surface water samples for related transformation products of nanomaterials has directly benefitted from the LTQ Orbitrap. The LC-LTQ-Orbitrap MS analysis was developed in part to provide a better screening technology for surface water samples that might contain these transformation products of nanomaterials [55].

Screen methods for emerging contaminants (new target compounds) in wastewater effluent, surface and ground water, along with finished drinking water can be reliably developed using the LTQ Orbitrap [39]. These newly developed work flows have demonstrated high-throughput sample processing using the most advanced LC-Orbitrap platform. A recent study indicated a rapid separation of targeted analytes within 14 min [56]. With the improved reliability for the detection of these emerging contaminants, the manufacturing companies and waste water treatment facilities will face stronger regulatory pressure for removal of these contaminants in the manufacturing processes. This is especially important for the hydrophilic compounds that are inherently more difficult to remove from complex mixtures [57].

## 4. Conclusions

The Orbitrap mass spectrometer with its high resolution and high mass accuracy capabilities has demonstrated that it is a powerful analytical tool in the investigation of the fate of environmental contaminants in complex matrices and mixtures [58]. The databases for identifying environmental contaminants continue to expand [41]. In addition, newly developed data analysis software and database search tools (e.g., spectral library searching, literature data) for the identification of emerging environmental contaminants in the aquatic environment aid in advancing the utility of this analytical platform [59]. The high resolution (*R* > 100,000) and accurate mass (mass error < 2 ppm) capabilities of the Orbitrap provide many new opportunities for applications in environmental research [60].

No one mass spectrometric technique is ideal for all applications in research in clinical and environmental settings. This technology is rapidly developing, and the future benefits are based upon the application, cost and performance desired. The FTMS technology has proven to be beneficial for many difficult analytical environmental questions. There are many different models of FTMS now available. While the FT-ICR is and will remain the ultimate standard for highest resolution MS and mass accuracy, the Orbitrap has proven to be more affordable and is highly applicable to answer many of these complex analytical/environmental questions. The ability to “dilute and shoot” may be one of the most important values of this FTMS technology. By diluting interfering substances, ionization is much less affected by enhancement or suppression. In this context, the U.S. Food and Drug Administration’s (FDA) Guidance for Industry: Bioanalytical Method Validation [61] recommends incurred sample reanalysis (ISR) in order to assess reproducibility, which also should be considered in environmental analyses. The use of FTMS is unparalleled in that role. High mass accuracy can eliminate questions of false positives that have been shown to exist with low resolution mass spectrometric methods [62]. In critical situations, FTMS can provide the necessary unambiguous answers through a combination of high resolution and high mass accuracy that analysts seek to provide.
